# Ocean Acidification and Warming Lead to Increased Growth and Altered Chloroplast Morphology in the Thermo-Tolerant Alga *Symbiochlorum hainanensis*

**DOI:** 10.3389/fpls.2020.585202

**Published:** 2020-11-17

**Authors:** Sanqiang Gong, Xuejie Jin, Yilin Xiao, Zhiyong Li

**Affiliations:** ^1^Marine Biotechnology Laboratory, State Key Laboratory of Microbial Metabolism and School of Life Sciences and Biotechnology, Shanghai Jiao Tong University, Shanghai, China; ^2^Key Laboratory of Tropical Marine Bio-Resources and Ecology, Guangdong Provincial Key Laboratory of Applied Marine Biology, South China Sea Institute of Oceanology, Chinese Academy of Sciences, Guangzhou, China; ^3^Southern Marine Science and Engineering Guangdong Laboratory, Guangzhou, China

**Keywords:** acidification, warming, thermophilic, *Symbiochlorum hainanensis*, coral reef algae

## Abstract

Ocean acidification and warming affect the growth and predominance of algae. However, the effects of ocean acidification and warming on the growth and gene transcription of thermo-tolerant algae are poorly understood. Here we determined the effects of elevated temperature (H) and acidification (A) on a recently discovered coral-associated thermo-tolerant alga *Symbiochlorum hainanensis* by culturing it under two temperature settings (26.0 and 32.0°C) crossed with two pH levels (8.16 and 7.81). The results showed that the growth of *S. hainanensis* was positively affected by H, A, and the combined treatment (AH). However, no superimposition effect of H and A on the growth of *S. hainanensis* was observed under AH. The analysis of chlorophyll fluorescence, pigment content, and subcellular morphology indicated that the chloroplast morphogenesis (enlargement) along with the increase of chlorophyll fluorescence and pigment content of *S. hainanensis* might be a universal mechanism for promoting the growth of *S. hainanensis*. Transcriptomic profiles revealed the effect of elevated temperature on the response of *S. hainanensis* to acidification involved in the down-regulation of photosynthesis- and carbohydrate metabolism-related genes but not the up-regulation of genes related to antioxidant and ubiquitination processes. Overall, this study firstly reports the growth, morphology, and molecular response of the thermo-tolerant alga *S. hainanensis* to future climate changes, suggesting the predominance of S. *hainanensis* in its associated corals and/or coral reefs in the future.

## Introduction

Ocean acidification and warming have led to shifts in seawater chemistry and carbonate saturation, which will potentially affect the physiology, behavior, and predominance of a range of organisms in marine ecosystems (Hoegh–Guldberg and Bruno, 2010). In recent decades, the mean pH value of surface seawater has declined by an average of 0.1 units, owing to the uptake of CO_2_ (Feely et al., 2009). Further decreases of 0.3–0.5 pH units and warming of 1–7°C are projected to occur by the end of this century (Feely et al., 2009; IPCC, 2014).

As primary producers, marine photosynthetic algae account for approximately half of global photosynthetic carbon fixation (Falkowski and Raven, 2013). Increasing evidence shows that ocean acidification influences the growth and/or gene transcription of algae, and the responses of algae to acidification are modulated by temperature, light, UV, and nutrient availability (Gao and Campbell, 2014; Gao et al., 2017; Boyd et al., 2018). For example, acidification may enhance the N_2_ fixation activity of cyanobacteria, but trace metal availability may neutralize or even reverse this effect (Zhang et al., 2019). Elevated CO_2_ enhances the growth of diatoms at low levels of sunlight but inhibits their growth at high levels (Gao et al., 2019). For most calcifying macroalgae, acidification under elevated solar UV and/or elevated temperature reduces their calcification (Jin et al., 2017; Martin and Hall-Spencer, 2017). Non-calcifying macroalgae, on the other hand, appear to benefit from elevated CO_2_ and show an enhanced growth rate (Gao et al., 1993; Cornwall et al., 2015, 2017). Furthermore, the geographic distribution and/or predominance of algae are also affected by future ocean acidification and warming conditions (Wernberg et al., 2011). A study of over 20,000 herbarium records of algae collected over 70 years from the Pacific and the Indian oceans around the Australian coast showed that a pole-ward shift of several temperate algal species is already occurring (Wernberg et al., 2011). Acquiring resistant Symbiodiniaceae from the environment or changing the relative abundance of Symbiodiniaceae associated with corals under elevated temperature have been reported (Baker et al., 2004; Fautin and Buddemeier, 2004). One field study provides evidence that acidification can lead to a predominance of macroalgae on reefs (Johnson et al., 2014). Those studies reveal that the response of algae to ocean acidification and warming are species specific, and certain thermophilic and/or stress-tolerant algae might have the ability to acclimate to future global climate changes and become predominant in the future. However, the individual and specifically combined effects of ocean acidification and warming on the growth and gene transcription of the thermophilic and/or stress-tolerant algae in the ocean remain poorly understood.

*Symbiochlorum hainanensis*, a recently discovered unicellular alga affiliated with Ulvophyceae, is widely associated with corals in the tropical coral reef areas of the South China Sea (SCS), where the annual average temperature is approximately 26°C (Gong et al., 2018, 2019). The optimal growth temperature of this alga is approximately 32°C (Supplementary Figure 1), which is higher than that of coral-symbiotic *Cladocopium* spp. (dominant Symbiodiniaceae associated with corals in the SCS; Supplementary Figure 2). Besides that, *S. hainanensis* can maintain rapid growth when it is cultured at 35°C (Supplementary Figure 1). Hence, *S. hainanensis* is a thermo-tolerant alga, and we propose that it may outcompete *Cladocopium* spp. and be the predominant species in coral–algae symbiont areas and/or coral reef areas in the future. However, how it will respond to future ocean acidification and warming conditions is unclear. Therefore, the present study performed a 28-day lab-scale experiment in which *S. hainanensis* was cultured under two temperature settings (∼26.0°C, *n* = 3 and ∼32.0°C, *n* = 3) crossed with two pH levels (∼8.16, *n* = 3 and ∼7.81, *n* = 3) to mainly explore the growth and the molecular-level response of *S. hainanensis* to the individual and combined effects of future ocean acidification and warming conditions.

## Materials and Methods

### Cultures and Experimental Setup

Cultures of *S. hainanensis* (CCTCC M2018096), isolated from coral species *Porites lutea* in the tropical reef regions of the SCS (Gong et al., 2018, 2019), were incubated in 250-ml Erlenmeyer flasks with 100 ml autoclaved artificial seawater medium (Formula grade A Reef Sea Salt, Formula, Japan) under an *in situ* temperature of 26°C, light intensity of 90 μmol photon m^–2^ s^–1^ with a 12-h/12-h light/dark cycle. The cultures were shaken at least three times a day. Cells in the mid-exponential phase were collected by centrifugation (5,000 rpm for 10 min) and washed three times with sterile phosphate-buffered saline. Then, cell pellets were re-suspended in autoclaved artificial seawater medium and used for further experiments.

Cultures of *S. hainanensis* were acclimated to four conditions: (1) *p*CO_2_ level 1,000 μatm (pH = 7.81), 26°C (A, *n* = 3), (2) *p*CO_2_ level 395 μatm (pH = 8.16), 32°C (H, *n* = 3), (3) *p*CO_2_ level 1,000 μatm (pH = 7.81), 32°C (AH, *n* = 3), and (4) *p*CO_2_ level 395 μatm (pH = 8.16), 26°C (C, *n* = 3). To achieve the different *p*CO_2_/pH conditions, 1,000-ml Erlenmeyer flasks with 500 ml autoclaved artificial seawater medium were bubbled with sterilized air containing either ambient (395 μatm, pH = 8.16) or elevated (1,000 μatm, pH = 7.81, Supplementary Figure 3) CO_2_ concentrations using outdoor air and CO_2_ chambers (HP1000G-D, China). To achieve the different temperature conditions, the Erlenmeyer flasks were incubated in water baths (26 or 32°C, Supplementary Figure 4). For each treatment, triplicate cultures were incubated under cool white fluorescent light intensity of 90 μmol photons m^–2^ s^–1^.

### Cell Growth and Morphology Observation

The cell concentration of *S. hainanensis* under the A (*n* = 3), H (*n* = 3), AH (*n* = 3), and C (*n* = 3) conditions was monitored every 2 days by optical density (OD) value at 750 nm with a UV spectrophotometer (UV-7504, China) at 750 nm. The growth rate was calculated based on the cell concentration variations of *S. hainanensis* at exponential phase (day 2 to day 24). The dry cell weight in the mid-exponential phase under the A (*n* = 3), H (*n* = 3), AH (*n* = 3), and C (*n* = 3) conditions was measured by filtering the algal suspension through a pre-dried and pre-weighted 0.45-μm cellulose nitrate membrane filter (Whatman, United States) and drying in an oven at 80°C for 24 h.

Chlorophyll fluorescence of *S. hainanensis* under the A (*n* = 3), H (*n* = 3), AH (*n* = 3), and C (*n* = 3) conditions was measured using a Turner fluorometer with the *in vivo* module (Trilogy, Turner Design, Sunnyvale, CA, United States). Algal cells in the mid-exponential phase under the A (*n* = 3), H (*n* = 3), AH (*n* = 3), and C (*n* = 3) conditions were sampled for chlorophyll *a*, chlorophyll b, and total carotenoids measurements according to the acetone-based method (Dere et al., 1998).

Cells of *S. hainanensis* in the mid-exponential phase under different conditions (A, H, AH, and C) were determined based on scanning electron and transmission electron microscopy observations according to our previous study (Gong et al., 2018).

### RNA Extraction, Purification, and Sequencing

Algal cells under the A (*n* = 3), H (*n* = 3), AH (*n* = 3), and C (*n* = 3) conditions were harvested in the mid-exponential phase by centrifugation at 5,000 rpm for 5 min at 4°C for total RNA extraction and RNA-seq. In detail, cell pellets under different conditions were snap-frozen in liquid nitrogen and stored at −80°C before RNA extraction. Total RNA was extracted from algal cells as previously described (Rosic and Hoegh–Guldberg, 2010). The RNA quantity and integrity were analyzed using a NanoDrop ND-1000 spectrometer (Wilmington, DE, United States) and an Agilent 2100 Bioanalyzer (Santa Clara, CA, United States). RNA samples with high purity (OD260/280 between 1.8 and 2.2) and high integrity (RNA integrity number > 8) were used for cDNA library construction. The size and the concentration of the cDNA libraries were determined by the Agilent 2100 Bioanalyzer (Santa Clara, CA, United States). All cDNA libraries were layered on a separate Illumina flow cell and sequenced using Illumina HiSeq 2000 (Illumina, Inc.). The raw sequence data produced in this study were deposited in the Sequence Read Archive (PRJNA662215) at the National Center for Biotechnology Information (NCBI).

### Quality Control and Short Read Assembly

Raw RNA-Seq reads under the A (*n* = 3), H (*n* = 3), AH (*n* = 3), and C (*n* = 3) conditions were processed using Trimmomatic v0.33 for trimming adapters as well as low-quality bases from the ends of the reads (Bolger et al., 2014). Poor-quality reads with average Phred quality score <20 and reads with lengths <55 were filtered out. The resulting set of good quality reads was then assembled with Trinity v2.1.1 software using default parameters (Grabherr et al., 2011; Haas et al., 2013). The assembly validation was performed using Bowtie2 aligner, where the filtered reads were mapped back to the assembled unigenes. Furthermore, non-redundant unigenes were retrieved with the aid of CD-HIT-EST software^1^ that clustered the unigenes with an identity parameter of 95%.

### Functional Annotation and Identification of Differentially Transcribed Genes

The *de novo*-assembled unigenes were searched against NCBI’s non-redundant protein and Swiss-Prot database using the BLASTX algorithm with an *e*-value cutoff of 10^–5^. Unigenes with significant matches were annotated using the Blast2GO platform (Conesa et al., 2005). Additional annotations were obtained through the Kyoto Encyclopedia of Genes and Genomes (KEGG) database through the KEGG Automatic Annotation Server (v1.6a) (Moriya et al., 2007). The cluster in the eggNOG database was employed to classify and group the putative and definitively identified proteins. The expression quantity of each unigene (fragments per kilobase of exon model per million mapped fragments) was estimated using RSEM (Li and Dewey, 2011). Differentially transcribed genes were selected using edge R as the method of choice (Robinson et al., 2010). Fold change differences were considered significant when a *P*-value <0.01 was achieved based on Benjamin and Hochberg’s false discovery rate procedure. A correlation analysis of differentially transcribed genes among different conditions was performed using the program package in the R software package (R 3.1.2). Bray–Curtis dissimilarity-based principal coordinate analysis (PCoA) pictures of differentially transcribed genes were drawn using Primer-e^2^ for comparing differentially expressed genes under different conditions.

### Quantitative Polymerase Chain Reaction

To validate the RNA-seq results, the expression level of 17 differentially transcribed genes under the A (*n* = 3), H (*n* = 3), AH (*n* = 3), and C (*n* = 3) conditions was measured using quantitative PCR (qPCR). The list of genes and primers is available in the electronic Supplementary Material (Supplementary Table 1). Complementary DNA was synthesized from 1 μg of total RNA, and qPCR was performed with an ABI ViiA 7 Real-Time PCR System (Applied Biosystems, United States) using FAST START SYBR green master mix according to the manufacturer’s instructions. The following procedure was used: 95°C for 10 min and one cycle for cDNA denaturation, 95°C for 10 s, 60°C for 20 s, 36 cycles for amplification, and one cycle for melting curve analysis (from 60 to 95°C) to verify the presence of a single product. qPCR was performed in triplicate for each sample. The relative expression levels were measured using Relative Expression Software Tool (REST).

### Statistical Analysis

All results are presented in the text as mean ± standard error. Significant differences (*P* < 0.01) in the OD value, dry cell weight, chlorophyll fluorescence, and pigment content under the A (*n* = 3), H (*n* = 3), AH (*n* = 3), and C (*n* = 3) conditions were tested by ANOVA using the stats package in R software (R Core Team, 2014). Significant (*P* < 0.01) differences in the Bray–Curtis distances of differentially transcribed genes under the A (*n* = 3), H (*n* = 3), AH (*n* = 3), and C (*n* = 3) conditions were determined by permutational multivariate analysis of variance (MANOVA) in Primer-e (see text footnote 2). Significant (*P* < 0.01) differences in qPCR-measured genes under the A (*n* = 3), H (*n* = 3), AH (*n* = 3), and C (*n* = 3) conditions were calculated using the pair-wise fixed reallocation randomization test in REST.

## Results

### Growth and Morphological Responses of *S. hainanensis* to H, A, and AH

**FIGURE 1:**
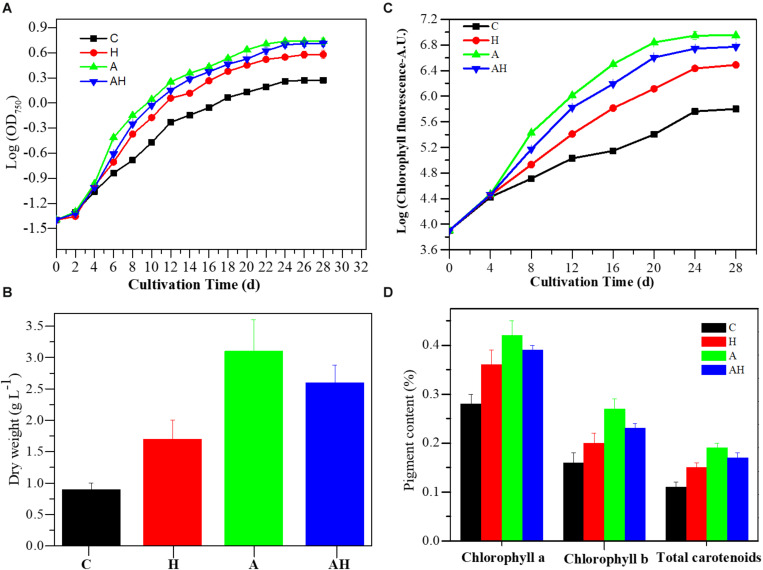
Growth characteristics of *Symbiochlorum hainanensis* in response to acidification and warming conditions. Growth curves **(A)**, cellular dry weight [**(B)** day 20], chlorophyll fluorescence **(C)**, and pigment content [**(D)** day 20] of *S. hainanensis* under the control (*in situ* temperature)—C (*n* = 3), elevated temperature—H (*n* = 3), acidification—A (*n* = 3), and combined treatment—AH (*n* = 3). All results are presented in the text as mean ± standard error.

The cell concentration, as measured by OD_750_ (Figure 1A) and cell dry weight (Figure 1B) of *S. hainanensis*, was significantly promoted by elevated temperature (H, *n* = 3), acidification (A, *n* = 3), and the combined treatment (AH, *n* = 3) compared with the control (C, *n* = 3; *P* < 0.01). The growth rate of *S. hainanensis* increased 1.05-fold but decreased 1.04-fold in the combined treatment compared with individual elevated temperature (AH-VS-H, *P* < 0.01) and acidification (AH-VS-A, *P* < 0.01), respectively (Supplementary Figure 5). In addition, chlorophyll fluorescence (Figure 1C) and pigment content (Figure 1D) showed similar variation trends with the algal growth of *S. hainanensis* under different conditions.

**FIGURE 2:**
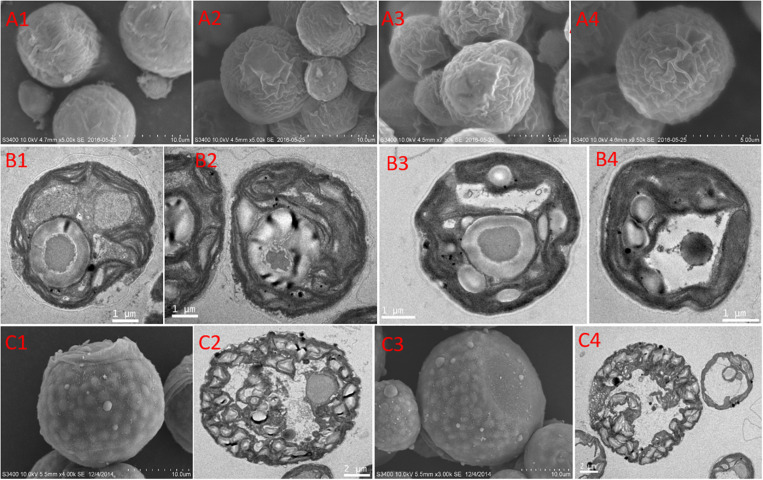
Morphological characteristics of *Symbiochlorum hainanensis* in response to acidification and warming. Scanning electron microscopic presentations of the vegetative cells of *S. hainanensis* under the control **(A1)**, elevated temperature **(A2)**, acidification **(A3)**, and combined treatment **(A4)**. Transmission electron microscopic presentations of vegetative cells of *S. hainanensis* under the control **(B1)**, elevated temperature **(B2)**, acidification **(B3)**, and combined treatment **(B4)**. Scanning and transmission electron microscopic presentations of aplanosporangiums of *S. hainanensis* under acidification conditions **(C1–C4)**.

The features of the cell morphology of *S. hainanensis* under different conditions were documented (Figure 2). The cell wall of *S. hainanensis* was furrowed (A2–A4), and the shape of the chloroplast changed (i.e., the volume of chloroplast became larger under acidification and warming conditions; B2–B4). More starch granules under acidification and warming conditions were observed (B2–B4). Moreover, the sporangium of *S. hainanensis* was observed under acidification condition (C1–C4).

### Transcriptomic Profiles of *S. hainanensis* Response to H, A, and AH

A total of 12 transcriptomic sequencing libraries were generated for the four conditions (C, H, A, and AH) with three biological replicates. These libraries were sequenced with an Illumina platform with an output of 88.36 G clean reads (an average of 7.36 G reads per sample) (Supplementary Table 2). The clean reads were pooled together and assembled into 95,827 unique genes, with an average length of 1,277 bp and a N50 value of 3,768 bp (Supplementary Table 3).

**FIGURE 3:**
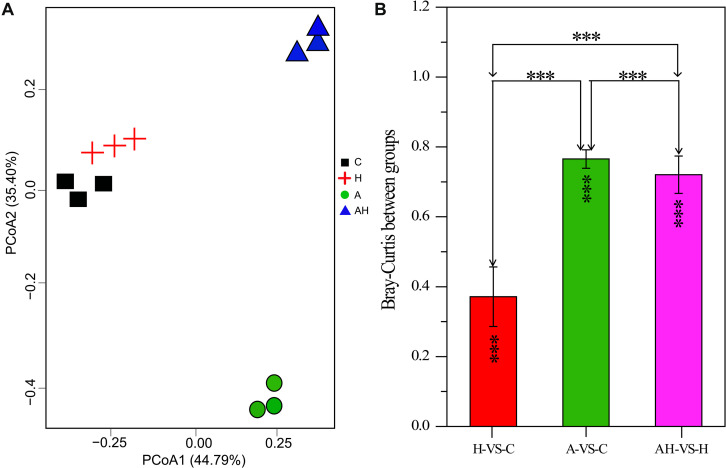
Plots of principal coordinate analysis **(A)** and permutational multivariate analysis of variance analysis **(B)** of differentially transcribed genes under different conditions: control—C (*n* = 3), elevated temperature—H (*n* = 3), acidification—A (*n* = 3), and combined treatment—AH (*n* = 3). Significant differences (*P* < 0.01) in Bray–Curtis distances of differentially transcribed genes under different conditions were determined using Primer-e. All results are presented in the text as mean ± standard error.

The PCoA analysis revealed that the gene transcription of *S. hainanensis* was significantly different under the C, H, A, and AH conditions (Figure 3, MANOVA, *P* < 0.001). The PCoA analysis showed the following order of the effect of different conditions on gene transcription: A-VS-C > AH-VS-H > H-VS-C. Among the detected genes, 688 genes were significantly differentially transcribed, and more than half of these genes had unknown functions (Supplementary File 1).

**FIGURE 4:**
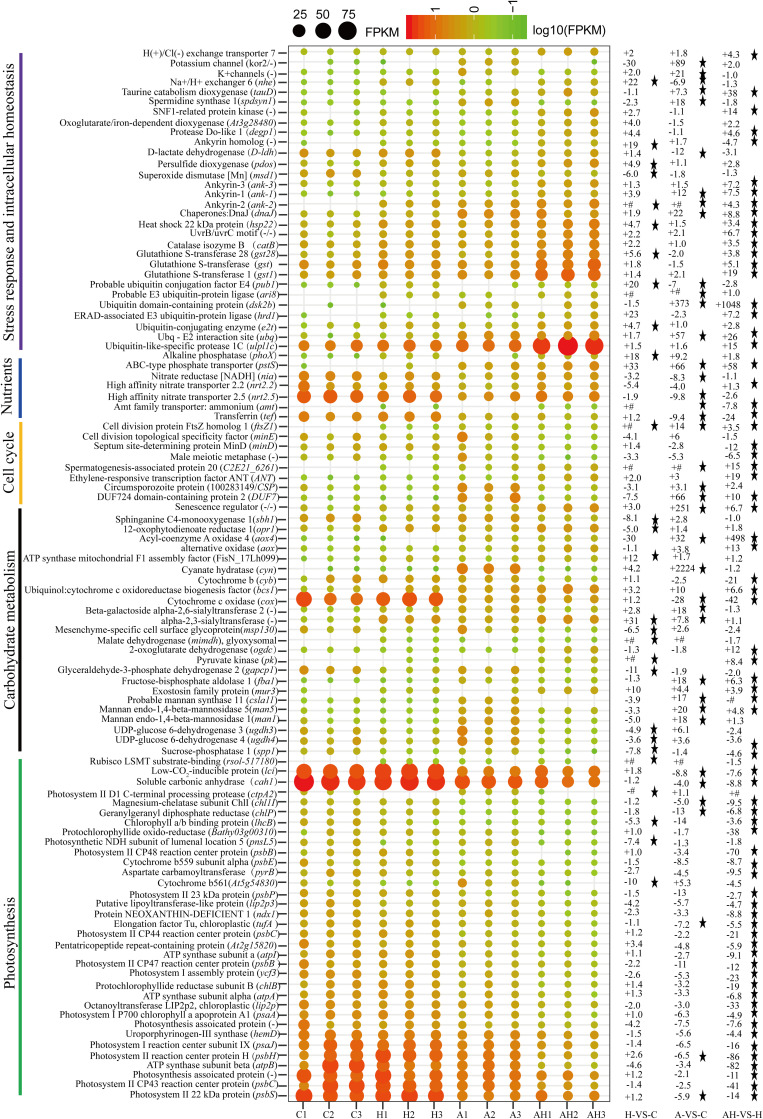
Profiles of differentially transcribed genes involved in key pathways under acidification and warming conditions. The control—C (*n* = 3), elevated temperature—H (*n* = 3), acidification—A (*n* = 3), and combined treatment—AH (*n* = 3). + signs represents up-regulated genes, – signs represent down-regulated genes, and star represents significantly changed genes under different conditions. All results are presented in the text as mean ± standard error.

The genes related to photosynthesis, CO_2_ biofixation, carbohydrate metabolism, cell cycle and control, nutrient input, transport and metabolism, stress response, and intracellular homeostasis were differentially transcribed under acidification and warming conditions (Figure 4). Other genes involved in flagella-related components, extracellular matrix (fasciclin-like protein, glycoprotein of tenascin, and the expansin superfamily of proteins), and transcriptional regulation also changed under different conditions (Supplementary File 1).

**FIGURE 5:**
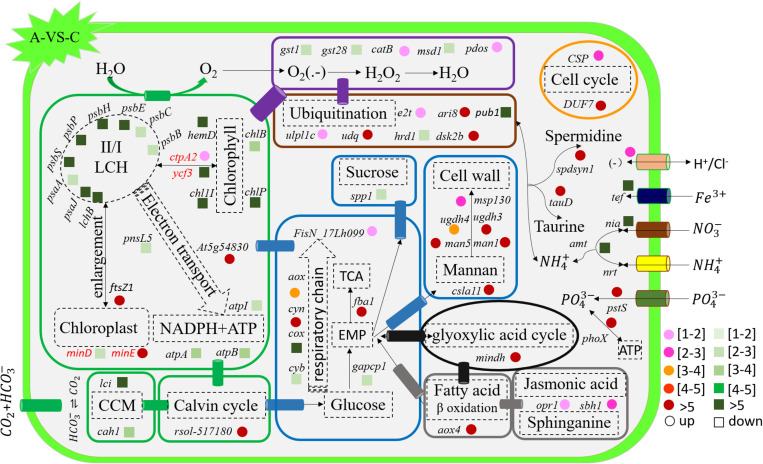
Schematic summary of the gene functions affected by acidification compared with the control (*n* = 3) of *in situ* temperature (*n* = 3). All results are presented in the text as mean ± standard error.

To validate the RNA-seq results, several key genes were selected for qPCR analysis. The results exhibited the same trends as the results of RNA-seq, confirming the reliability of our present analysis (Supplementary Table 1).

### Photosynthesis-Related Gene Responses to H, A, and AH

**FIGURE 6:**
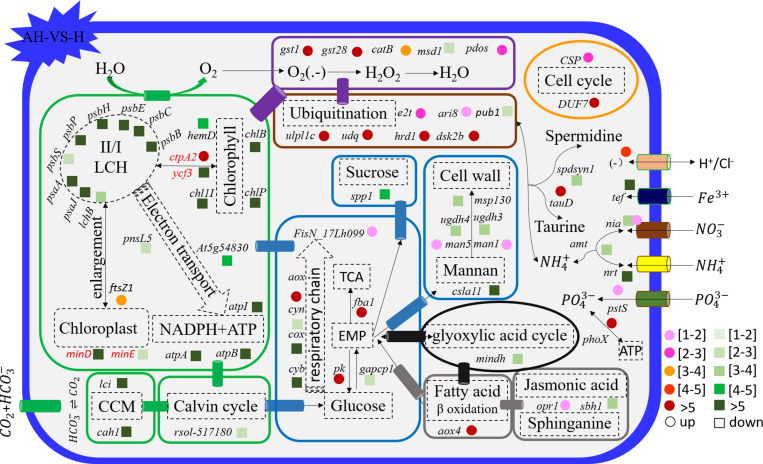
Schematic summary of the gene functions affected by the combined treatment (*n* = 3) compared with elevated temperature (*n* = 3). All results are presented in the text as mean ± standard error.

The transcription of genes related to photosynthesis was minimally affected by elevated temperature (H-VS-C) (Figure 4), while gene transcription was obviously affected by acidification (A-VS-C) and the combined treatment (AH) (Figures 4–6). Specifically, nearly all differentially transcribed genes related to photosynthesis were significantly down-regulated under the combined treatment. For example, the genes of *psaA*, *psaJ*, and *ycf3* involved in photosystem I, *psbB*, *psbC*, *psbE*, *psbH*, *psbP*, and *psbS* involved in photosystem II, *lhcB*, *chlB*, *chlP*, and *chl11* involved in light-harvesting complex (LHC) protein, and *atpA* and *atpB* involved in photosynthetic ATP synthesis were down-regulated more than 1–15-fold by the combined treatment (AH) (Figures 4, 6). In addition, the *lci* and *cah1* genes encoding low CO_2_-inducible protein and soluble carbonic anhydrase related to carbon acquisition by CO_2_ concentrating mechanism (CCM) were significantly down-regulated more than fourfold by individual acidification and the combined treatment (Figures 4–6).

### Carbohydrate Metabolism-Related Gene Responses to H, A, and AH

Acidification and elevated temperature had different effects on the genes related to the carbohydrate metabolism of *S. hainanensis* (Figures 4–6). Almost all the differentially transcribed genes involved in carbohydrate metabolism were up-regulated by acidification (A-VS-C), while most were minimally affected by elevated temperature and the combined treatment (both H-VS-C and AH-VS-H). Compared with the control (C), individual acidification (A) caused the genes *fba*, *clsa11*, *ugdh3*, *ugdh4*, *man1*, *man5*, *msp130*, *mindh*, and *aox4* related to the citric acid cycle (TCA), cell wall polysaccharide synthesis, and β oxidation of fatty acids to be significantly up-regulated more than threefold. However, the transcription of those genes was down-regulated approximately 1–17-fold by the combined treatment (AH).

### Cell and Life Cycle-Related Gene Responses to H, A, and AH

Elevated temperature (H) also had a minimal effect on the transcription of genes involved in the cell and life cycles. However, almost all of these genes were up-regulated by individual acidification (A-VS-C), while most of them were down-regulated by the combined treatment (AH-VS-H) (Figures 4–6). Genes encoding DUF724 domain-containing protein and circumsporozoite protein related to sporulation were up-regulated by both individual acidification (A) and the combined treatment (AH). Specifically, the gene encoding DUF724 domain-containing protein was up-regulated approximately 66-fold by individual acidification (A). In addition, the key gene *ftsZ*, which is involved in chloroplast division, was significantly up-regulated by A, H, and AH compared with the control (C).

### Nutrient Transport- and Metabolism-Related Gene Responses to H, A, and AH

The transcription of genes related to nutrient transport and metabolism is illustrated in Figures 4–6. Genes encoding alkaline phosphatase and ABC-type phosphate transporter involved in phosphate metabolism were up-regulated more than 10-fold, whereas genes encoding NO_3_^–^ transporter and nitrate reductase involved in nitrogen metabolism were down-regulated over threefold under elevated temperature (H-VS-C). Under the combined treatment (AH), genes involved in nitrogen and phosphate metabolism were up-regulated over threefold relative to the acidification treatment (A). In addition, the gene encoding transferrin involved in Fe^3+^ transporter showed a higher transcription under the control (C) and elevated temperature (H) conditions than under the other conditions, and it was significantly down-regulated approximately fivefold by acidification (A) and the combined treatment (AH).

### Stress- and Intracellular Homeostasis-Related Gene Responses to H, A, and AH

Genes encoding glutathione S-transferase, catalase isozyme, UvrB/uvrC motif, heat shock 22-kDa protein, and chaperones were differentially transcribed under single acidification (A) or elevated temperature condition (H) (Figures 4–6). Specifically, the transcription of these genes was up-regulated more than twofold by the combined treatment (AH) than by individual acidification (A), except for one gene encoding chaperone DnaJ. Moreover, genes related to ubiquitination processes were highly transcribed under different conditions.

## Discussion

In this study, we mainly explored the growth, morphology, and molecular level response of *S. hainanensis*, a recently discovered coral-associated thermo-tolerant alga, to future ocean acidification and warming conditions by culturing it under two temperature settings (∼26.0 and ∼32.0°C) crossed with two pH levels (∼8.16 and ∼7.81) in a 28-day lab-scale experiment. We found that this thermo-tolerant alga exhibited a positive growth response to individual acidification, elevated temperature, and the combined treatment. We observed a no-superimposition effect of individual acidification and elevated temperature on the growth of *S. hainanensis* compared with the combined treatment of acidification and elevated temperature. Interestingly, our present data indicated that the chloroplast enlargement (possibly controlled by the *ftsZ* gene) along with the increase of chlorophyll fluorescence and pigment content might be a universal mechanism for the stimulative growth of *S. hainanensis* under ocean acidification and warming conditions, implying the predominance of *S. hainanensis* in its associated corals and/or coral reef areas in the future. This study provides novel insights into the growth, morphology, and molecular-level responses of thermo-tolerant algae to ocean warming and acidification conditions.

The stimulative growth response of *S. hainanensis* to ocean acidification reported in this study is consistent with the previous studies in several species of cyanobacteria, diatoms, and dinoflagellates (Brading et al., 2011; Wu et al., 2014; Walworth et al., 2016; Huang et al., 2018). The enhanced growth may be attributed to more carbon resources and down-regulated CCMs (an efficient process needs energy and resources for CO_2_ fixation by algae) caused by the increasing partial pressure of CO_2_ (*p*CO_2_) in seawater under acidification (Riebesell et al., 1993; Tortell et al., 2008). Our transcriptomic results also showed that two genes encoding low CO_2_-inducible protein (*lci*) and soluble carbonic anhydrase (*cah1*) associated with CCMs were significantly down-regulated by acidification. Although the genes encoding RubisCO for CO_2_ fixation were not differentially transcribed in *S. hainanensis* under acidification, one gene encoding RubisCO LSMT was significantly up-regulated. RubisCO LSMT was reported to exhibit methyltransferase activity toward RubisCO (Ying et al., 1999); thus, we propose that the up-regulation of the gene encoding RubisCO LSMT might be involved in the regulation of CO_2_ fixation in *S. hainanensis* under acidification. In addition, we observed that acidification enhanced the transcription of genes related to cell activity and growth (i.e., sucrose and cell wall polysaccharide synthesis, EMP, TCA, glyoxylic acid cycle, β-oxidation of fatty acid, and cyanide-resistant respiration), which might contribute to the enhanced growth of *S. hainanensis* as well. Similar results were also reported in higher plants under elevated CO_2_ or stresses (Solomos, 1977; Li et al., 2008). Notably, our results showed that almost all genes involved in photosynthesis (i.e., *psbB-psbS* of photosynthetic II, *psaA* and *psaJ* of photosynthetic I, and *lchB* of LHC) were down-regulated by acidification. According to one previous study of diatom (Goldman et al., 2017), a likely reason for the variability in the response of *S. hainanensis* (excluding potential modulation by other environmental factors, i.e., temperature) to acidification is that the increasing *p*CO_2_ (carbon resource) and the concomitantly decreasing pH (acidification) separately have different effects on the growth and photosynthesis. Interestingly, we observed that the chlorophyll fluorescence and pigment content of *S. hainanensis* significantly increased under acidification. Concomitantly, enlargement of chloroplast and significant up-regulation of one key gene *ftsZ* (encoding cell division protein homolog 1) for controlling chloroplast division and/or morphogenesis (Strepp et al., 1998; Wang et al., 2002) were observed under acidification in *S. hainanensis*. Therefore, we speculated that the significant up-regulation of *ftsZ* gene might contribute to the enlargement of chloroplast and the enhancement of chlorophyll fluorescence and pigment content of *S. hainanensis* under acidification.

The present results showed that the growth of *S. hainanensis* was greatly promoted by an elevated temperature of 32°C. To our knowledge, this was the first report of a positive response of marine algae to an elevated temperature up to 32°C. A previous study suggested that marine algae may exhibit much lower optimal growth temperature than tropical seagrasses and macroalgae which showed optimal growth temperature ranging from 27 to 33°C (Koch et al., 2013). Biber (2007) reported that the biomass of Florida algae significantly decreased when the temperatures were above 31°C. Fleshy, branching tropical macroalgae species maintain relatively consistent net productivity rates at 32°C (Mejia et al., 2012). The photosynthesis of the tropical *Codium edule* (macroalgae) was disrupted and inhibited at 32 and 35°C, respectively (Lee and Hsu, 2010). Even for the generally considered thermo-tolerant Symbiodiniaceae (*Durusdinium* spp.) associated with corals, both net growth and negative effect of photosynthesis at 32°C were reported (Karim et al., 2015). Notably, our results showed that *S. hainanensis* isolated from tropical reefs in the South China Sea exhibited a high optimal growth temperature of 32°C, which was higher than those of previously reported algal species. In reality, *S. hainanensis* could maintain a rapid growth even when it was cultured at 35°C. The transcriptomic results showed that, in addition to up-regulating genes involved in protein folding, oxidative stress, and ubiquitination processes, elevated temperature had a minimal effect on the transcription of genes involved in the basic metabolism of *S. hainanensis*, such as photosynthesis, carbohydrate metabolism, the cell cycle, and nutrient metabolism. As what previous studies have suggested for other organisms (Kumar et al., 2017; Ruocco et al., 2017), the up-regulation of genes related to protein folding (i.e., heat shock protein and chaperone) might contribute to the thermal acclimation of *S. hainanensis.* It is widely believed that the production of reactive oxygen species increases under elevated temperatures or other stresses, and plants/algae must activate their antioxidant defense mechanisms to protect themselves from oxidative damage (Ledford and Niyogi, 2005; Lazaro et al., 2016). Similarly, genes encoding glutathione S-transferase, catalase, and persulfide dioxygenase related to antioxidant defense mechanisms were up-regulated in *S. hainanensis*. The ubiquitination processes may also have aided in the ability of this alga to acclimate to high temperatures given the up-regulation in the transcription of the respective genes, such as up-regulating genes encoding ubiquitin ligase (over 23-fold) (Mayfield et al., 2014). In addition, the increase of chlorophyll fluorescence and pigment content, the enlargement of chloroplast, and the significant up-regulation of *ftsZ* gene encoding cell division protein homolog 1 were also observed under elevated temperature in *S. hainanensis*, which might also contribute to the enhanced growth of *S. hainanensis* under elevated temperature.

Laboratory studies have shown that the effects of the combined treatment of elevated temperature and acidification on the growth of marine algae are species specific (Hyun et al., 2014; Gao et al., 2019). For example, the growth of the picoplanktonic cyanobacterium *Synechococcus* was promoted by the combined treatment, whereas elevated temperature and acidification had no effects on *Prochlorococcus* (Fu et al., 2007). Similarly, the combined treatment promoted the growth of the diatom *Skeletonema* (Kremp et al., 2012), but it had no obvious effects on *Thalassiosira* and *Chaetoceros* (Hyun et al., 2014). The growth of coccolithophore *E. huxleyi* was inhibited by the combined treatment (Listmann et al., 2016). Our present results indicated that the combination of elevated temperature and acidification (AH) had a positive effect on the growth of *S. hainanensis* (AH-VS-C). Meanwhile, we observed no superimposition effect on the growth of *S. hainanensis* in response to acidification and elevated temperature alone, compared to the combined treatment.

For transcriptional profiles, the effects of individual acidification and warming conditions on the response of marine algae and terrestrial plant have been widely reported (Li et al., 2008; Kumar et al., 2017; Ruocco et al., 2017). To our knowledge, however, data on the effect of the combined treatment of elevated temperature and acidification (AH) on marine algae have not been reported, and we do not know the mechanisms involved. Differently from the no-superimposition effect in the growth of *S. hainanensis*, a superimposition effect of the individual elevated temperature (H) and acidification (A) on the gene transcription of *S. hainanensis* was observed under combined treatment (AH). Meanwhile, our present transcriptomic data revealed a balanced strategy used by *S. hainanensis* for maintaining moderate growth under the combined treatment of elevated temperature and acidification (AH). For example, the transcription of genes related to photosynthesis, CO_2_ biofixation, and carbohydrate metabolism was inhibited, but genes related to antioxidant and ubiquitination processes were promoted under combined treatment (AH) compared with individual elevated temperature (H) and acidification (A).

## Conclusion

This study mainly describes the growth, morphology, and molecular response of *S. hainanensis*, a recently discovered coral-associated thermo-tolerant alga, to future ocean acidification and warming conditions. The promoted growth of *S. hainanensis* under these conditions suggests the strong acclimation of this alga to future ocean environmental changes. An antagonistic effect on the growth of *S. hainanensis* was observed between elevated temperature and acidification, which are involved in the balance of gene transcription related to basic metabolism and stress responses. The present data revealed that chloroplast morphogenesis (possibly controlled by *ftsZ* gene) along with increasing chlorophyll fluorescence and pigment content might serve as a universal mechanism for promoting the growth of this alga under both acidification and warming conditions. This alga is also ecologically important because it is highly abundant in corals in the SCS, and the optimum growth temperature of 32°C for *S. hainanensis* isolated from bleached corals in the SCS is higher than those of the symbiotic *Cladocopium* spp. and *Durusdinium* spp. (thermal-sensitive and thermal-tolerant Symbiodiniaceae majorly associated with corals in the SCS). The present study provides novel and valuable data on the growth of *S. hainanensis* and improves the knowledge of the molecular functions underpinning the growth response of thermo-tolerant algae to future ocean acidification and warming. This study also lays a foundation for a further evaluation of the distribution and the ecological significance of *S. hainanensis* in coral–algae symbionts and/or coral reef areas.

## Data Availability Statement

The datasets presented in this study can be found in online repositories. The names of the repository/repositories and accession number(s) can be found in the article/Supplementary Material.

## Author Contributions

SG and ZL conceived and designed the experiments. SG and YX performed the cultivation experiment and sampling. SG performed the molecular and bioinformatic analyses. SG, XJ, and ZL wrote the manuscript. All the authors read and approved the final manuscript.

## Conflict of Interest

The authors declare that the research was conducted in the absence of any commercial or financial relationships that could be construed as a potential conflict of interest.

## Footnotes

1 http://weizhongli-lab.org/cd-hit/

2 http://www.primer-e.com/
