# Supplementary material for: Ocean Acidification and Warming Lead to Increased Growth and Altered Chloroplast Morphology in the Thermo-Tolerant Alga Symbiochlorum hainanensis
Source: Front Plant Sci. 2020 Nov 17;11:585202. doi: 10.3389/fpls.2020.585202 (PMC7705064; doi:10.3389/fpls.2020.585202)
Supplement: Supplementary file 2 [file Table_2.DOCX]

**Supplementary Data for:**

**Ocean acidification and warming lead to increased growth and altered chloroplast morphology in the thermo-tolerant algal *Symbiochlorum hainanensis***

Sanqiang Gong^1,2,3^, Xuejie Jin^2,3^, Yilin Xiao^1^, Zhiyong Li^1*^

^1^Marine Biotechnology Laboratory, State Key Laboratory of Microbial Metabolism and School of Life Sciences & Biotechnology, Shanghai Jiao Tong University, 800 Dongchuan Road, Shanghai 200240, China

^2^Key Laboratory of Tropical Marine Bio-resources and Ecology & Guangdong Provincial Key Laboratory of Applied Marine Biology, South China Sea Institute of Oceanology, Chinese Academy of Sciences, Guangzhou, China

^3^Southern Marine Science and Engineering Guangdong Laboratory (Guangzhou), P.R. China

*****Corresponding author: Zhiyong Li, TEL: 86 02134204036; E-mail:[zyli@sjtu.edu.cn](mailto:zyLi@sjtu.edu.cn);

**Contents:**

**Supplementary Table S1.**

| **Gene** | **Primer sequence (5’→ 3’)** | **Product size (bp)** | **log2 relative expression (Transcriptome)** | **log2 relative expression (qPCR)** |
| --- | --- | --- | --- | --- |
| *psbS* | Fw:ATTGTTTCGCCGATGAGGCT | 123 | -3.47 | -2.43 |
|  | Rv:CTCGCTCTTGCAGCCTAAGA |  |  |  |
| *psaJ* | Fw:GAAGGCATGCATGGACGTAAC | 143 | -4.56 | -6.47 |
|  | Rv:CCGAAAGCTCAGCTGCAGTAAT |  |  |  |
| *atpB* | Fw:ACACGGCGGTGTTTCTGTAT | 160 | -8.71 | -9.22 |
|  | Rv:TGCACCAGGTGGTTCATTCA |  |  |  |
| *psbC* | Fw:CTTCATGGCATGCGCAACTT | 121 | -5.95 | -3.26 |
|  | Rv:TGTGTCCCATAGTCCGTTGC |  |  |  |
| *minE* | Fw:GTGCAGCTTTGTGGGAAAGG | 132 | -4.13 | -5.23 |
|  | Rv:GCACGAATCGACAACAAGCA |  |  |  |
| *ftsZ1* | Fw:CGTGTCTGAAGTCGTCACCA | 102 | 8.04 | 11.03 |
|  | Rv:ATCAGCGTGACCTGGATCTC |  |  |  |
| *cah1* | Fw:CCAGTGGAACTGGATGGGTG | 179 | -3.5 | -4.29 |
|  | Rv:GCACCATCGGCAATGTATCC |  |  |  |
| *rsol-517180* | Fw:CCAACGTGGTGTTCAAGCTG | 135 | 9.06 | 4.57 |
|  | Rv:AGTCGATCATCGGGCACATC |  |  |  |
| *pox4* | Fw:TTGTGACAGACGAGTGGGTG | 110 | 3.96 | 2.11 |
|  | Rv:TTTCAGTCGCGCAAACACAG |  |  |  |
| *mimd*h | Fw:GCTACGTGTCCTGAAAGGGT | 104 | 7.79 | 9.22 |
|  | Rv:AGAGACAACTGCGACTCACC |  |  |  |
| *cat* | Fw:GCGTTCGGGAATCCTCTCTC | 175 | 2.88 | 1.56 |
|  | Rv:CAAGTACCGACCGAGTGCAA |  |  |  |
| *hsp90* | Fw:GTTAACAGCTGGCACTGGGT | 103 | 8.67 | 6.78 |
|  | Rv:TAAGATCTCCCGCGTCCAAC |  |  |  |
| *clcn7* | Fw:TGCTCCCTCCAACTGAGGAT | 183 | 3.14 | 4.12 |
|  | Rv:GGAAGGGAGCATCGAATCGT |  |  |  |
| *amt* | Fw:GGGGTTCGTTTCCATCGACT | 132 | 8.63 | 7.99 |
|  | Rv:CTTTCTCTGCGCGTTTTGCT |  |  |  |
| *mfs* | Fw:ATCACAACACGGGCGAAGAT | 107 | -3.3 | -4.65 |
|  | Rv:GCTGCCTTGGGACCTGTTAT |  |  |  |
| *tf* | Fw:TCACCAACTCTCGTGACTGC | 172 | -4.33 | -3.12 |
|  | Rv:AGATCGTGGGGGTTATTGCC |  |  |  |
| *corA* | Fw:CAAACGACGTTAACCCGGTG | 114 | -8.62 | -8.7 |
|  | Rv:CGTCCATGATCTCCTCCAGC |  |  |  |
| *ef1* | Fw:TGGGTGCTGGACAACCTCAA | 186 | - | - |
|  | Rv:TCCAGATGCCACCACGAGAA |  |  |  |

**Supplementary Table S2.** **Summary of NGS data**

| Samples | Raw reads | Clean reads | Clean bases | Q30 (%) | Q30 (%) | GC (%) |
| --- | --- | --- | --- | --- | --- | --- |
| C1 | 48947436 | 48496566 | 7.22G | 96.73 | 91.64 | 60.12 |
| C2 | 49392910 | 48603294 | 7.18G | 96.46 | 91.25 | 58.23 |
| C3 | 51066962 | 50258680 | 7.44G | 96.30 | 90.87 | 58.54 |
| H1 | 50810968 | 49950510 | 7.39G | 96.22 | 90.74 | 57.85 |
| H2 | 51340218 | 48859422 | 7.28G | 96.71 | 91.54 | 60.82 |
| H3 | 51011302 | 50292996 | 7.46G | 96.17 | 90.57 | 59.14 |
| A1 | 49811892 | 49251268 | 7.32G | 96.51 | 91.19 | 60.61 |
| A2 | 51634962 | 51026082 | 7.57G | 96.69 | 91.64 | 59.87 |
| A3 | 50646144 | 50020898 | 7.41G | 96.63 | 91.49 | 60.31 |
| AH1 | 51223192 | 50657822 | 7.52G | 96.67 | 91.56 | 60.59 |
| AH2 | 47951434 | 47472094 | 7.06G | 96.67 | 91.53 | 60.51 |
| AH3 | 49294004 | 50654132 | 7.51G | 96.44 | 91.13 | 59.20 |

**Supplementary Table S3.** **Summary of de novo-assembled unigenes**

| Type | sequences | bases | Min_len | Max_len | Average_len | N50 | GC (%) |
| --- | --- | --- | --- | --- | --- | --- | --- |
| Unigenes | 95827 | 122Mb | 201bp | 24927bp | 1277bp | 3768bp | 61.5 |

**Supplementary Figure S1. The effect of temperature on the growth of *Symbiochlorum hainanensis*.** All results are presented in text as mean ± standard error (SE).

**Supplementary Figure S2. The effect of temperature on the growth of *S. hainanensis* and *Cladocopium sp.*.** All results are presented in text as mean ± standard error (SE).

**
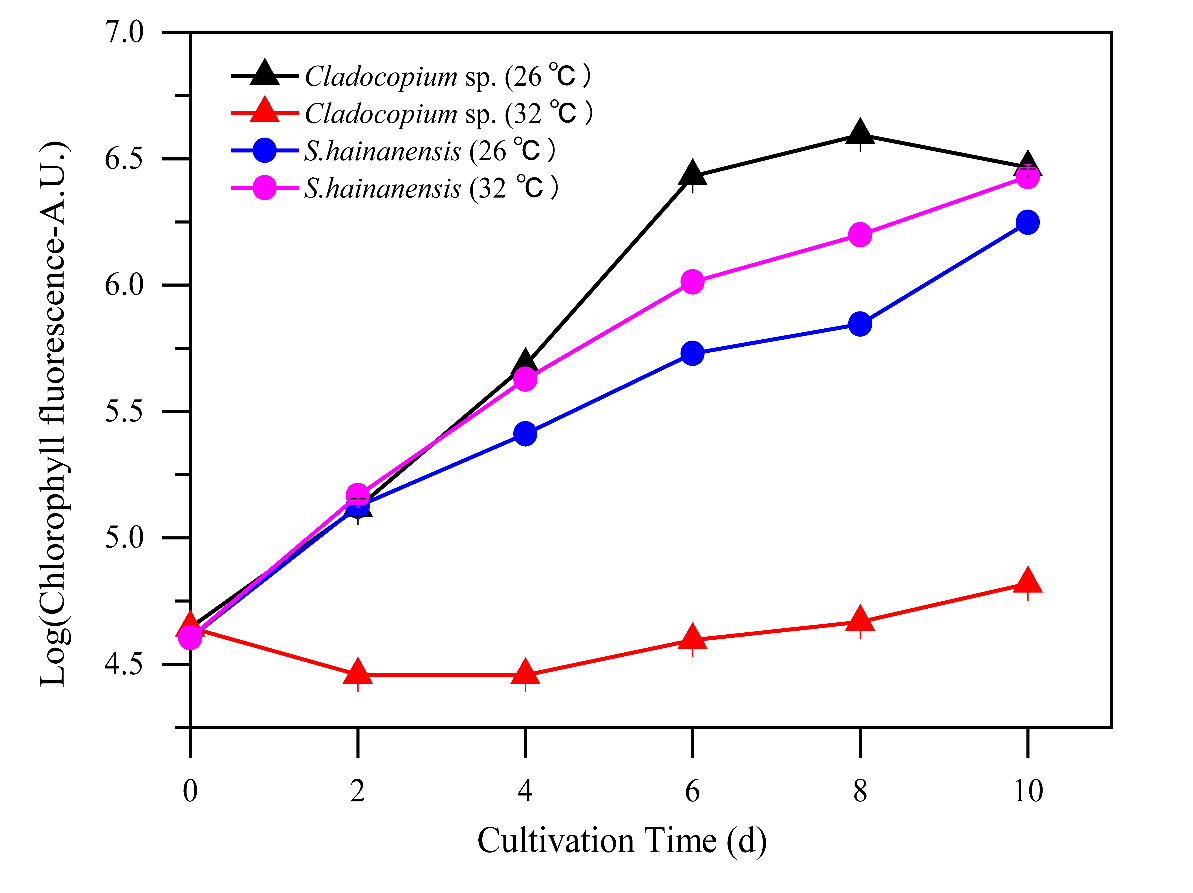
**

**Supplementary Figure S3. The changes of pH during cultivation of *Symbiochlorum hainanensis* under different conditions.** The control (in situ temperature)-C (n=3), elevated temperature-H (n=3), acidification-A(n=3) and combined treatment-AH (n=3). All results are presented in text as mean ± standard error (SE).

**
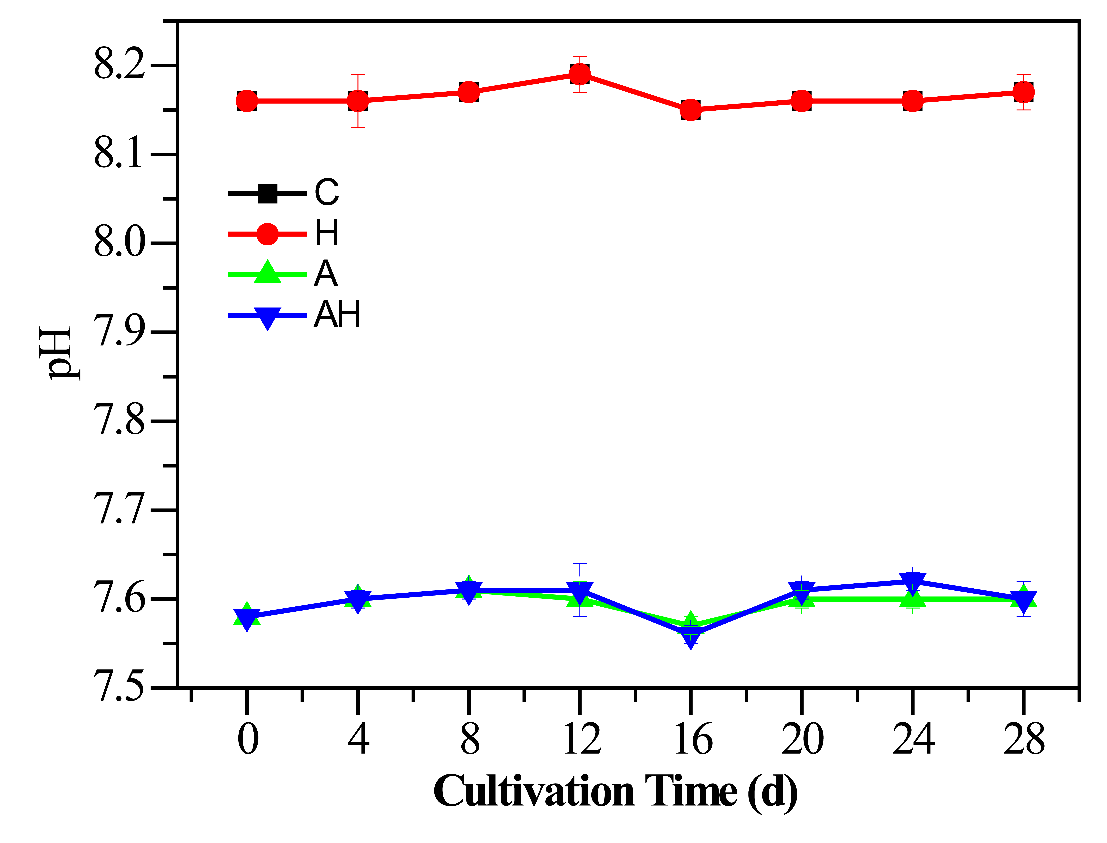
**

**Supplementary Figure S4.** **The changes of temperature during cultivation of *Symbiochlorum hainanensis* under different conditions.** The control (in situ temperature)-C (n=3), elevated temperature-H (n=3), acidification-A(n=3) and combined treatment-AH (n=3). All results are presented in text as mean ± standard error (SE).

**
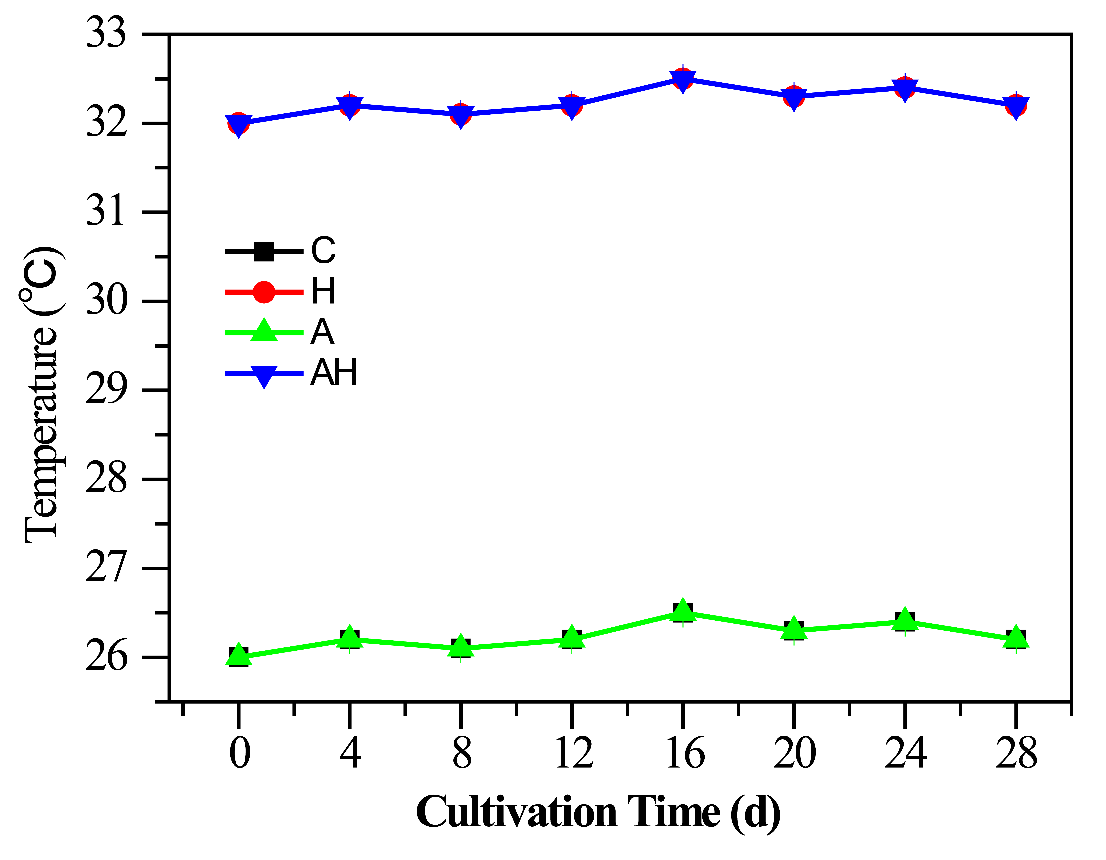
**

**Supplementary Figure S5. Growth rates of *S. hainanensis* in response to acidification and warming conditions*.*** The control (in situ temperature)-C (n=3), elevated temperature-H (n=3), acidification-A(n=3) and combined treatment-AH (n=3). All results are presented in text as mean ± standard error (SE).
